# Multiple Sclerosis Susceptibility-Associated SNPs Do Not Influence Disease Severity Measures in a Cohort of Australian MS Patients

**DOI:** 10.1371/journal.pone.0010003

**Published:** 2010-04-02

**Authors:** Cathy J. Jensen, Jim Stankovich, Anneke Van der Walt, Melanie Bahlo, Bruce V. Taylor, Ingrid A. F. van der Mei, Simon J. Foote, Trevor J. Kilpatrick, Laura J. Johnson, Ella Wilkins, Judith Field, Patrick Danoy, Matthew A. Brown, for the The Australian and New Zealand Multiple Sclerosis Genetics Consortium (ANZgene), Justin P. Rubio, Helmut Butzkueven

**Affiliations:** 1 Howard Florey Institute, Melbourne, Australia; 2 Physiology Department, Faculty of Medicine, Nursing and Health Sciences, Monash University, Melbourne, Australia; 3 Menzies Research Institute, University of Tasmania, Hobart, Australia; 4 The Royal Melbourne Hospital, Melbourne, Australia; 5 Centre for Neuroscience, University of Melbourne, Melbourne, Australia; 6 The Box Hill Hospital, Box Hill, Victoria, Australia; 7 The Walter and Eliza Hall Institute of Medical Research, Melbourne, Australia; 8 Diamantina Institute of Cancer, Immunology and Metabolic Medicine, Princess Alexandra Hospital, University of Queensland, Brisbane, Queensland, Australia; 9 Botnar Research Centre, Nuffield Department of Orthopaedic Surgery, University of Oxford, Oxford, United Kingdom; Julius-Maximilians-Universität Würzburg, Germany

## Abstract

Recent association studies in multiple sclerosis (MS) have identified and replicated several single nucleotide polymorphism (SNP) susceptibility loci including *CLEC16A*, *IL2RA*, *IL7R*, *RPL5*, *CD58*, *CD40* and chromosome 12q13–14 in addition to the well established allele HLA-DR15. There is potential that these genetic susceptibility factors could also modulate MS disease severity, as demonstrated previously for the MS risk allele HLA-DR15. We investigated this hypothesis in a cohort of 1006 well characterised MS patients from South-Eastern Australia. We tested the MS-associated SNPs for association with five measures of disease severity incorporating disability, age of onset, cognition and brain atrophy. We observed trends towards association between the RPL5 risk SNP and time between first demyelinating event and relapse, and between the CD40 risk SNP and symbol digit test score. No associations were significant after correction for multiple testing. We found no evidence for the hypothesis that these new MS disease risk-associated SNPs influence disease severity.

## Introduction

Many familial and population based studies have demonstrated the role of genetic factors in susceptibility to multiple sclerosis (MS). By far the most important and consistently replicated genetic risk factor associated with MS is the HLA- DRB1*1501-DQB1*0602 (HLA-DR15) haplotype [1] which carries an odds ratio of between 2.0 and 3.5 for MS [2].

In the last two years, a series of genome-wide association studies have identified other SNPs that are associated with multiple sclerosis. Some of the recent discoveries are polymorphisms in genes for interleukin 2 receptor alpha (*IL2RA*), interleukin 7 receptor (*IL7R*), C-type lectin, family 16, member A (*CLEC16A*), ribosomal protein L5 (*RPL5*)/*EV15* locus, *CD40*, and rs703842 on chromosome 12 [1], [3], [4], [5], [6]. Individually, these SNPs contribute a small proportion to overall MS risk, with allelic odds ratios of between 1.1–1.3 [1], [3], [4], [5], [6].

As well as influencing MS risk, the HLA-DR15 haplotype has been reported to reduce the age of MS onset in MS patients in a number of large, independent studies from Scandinavia and Britain [2], [7], [8], [9], [10] although this was not replicated in a large US study [11]. Interestingly, in one of these studies [2], the HLA-DR15 allele was significantly more frequent in patients with definite MS than in patients with possible or probable MS (defined according to the Poser criteria) which may be a proxy for severity. In another study HLA-DR2 (which includes the HLA-DR15 risk allele), was associated with increased risk of a more severe course of disease [12]. In addition HLA-DR15 positivity has been associated with increased lesion load at MS onset [13]. At the most benign end of the wide phenotypic MS spectrum, it may be an entirely subclinical disease, exemplified in twin studies, in which 13% percent of asymptomatic monozygotic twins and 9% of asymptomatic dizygotic twins of MS index cases exhibited cerebral MRI lesions typical of MS [14]. It is therefore possible that MS susceptibility alleles actually represent determinants of severity, acting at the mildest end of the MS spectrum, by increasing the likelihood of clinically overt disease and thus, diagnosis. If this were the case, and if genetic modulation of MS severity were present across the clinical spectrum of disease, one might also be able to detect an influence of risk SNPs on MS phenotype, manifesting as an increased disease severity in MS patients carrying the risk SNPs.

In order to test this hypothesis, we have chosen to examine the potential contribution of the susceptibility SNPs in *IL2RA*, *IL7R*, *CLEC16A*, *RPL5*, *CD40*, *CD58* and the Chr12 loci to MS severity, using a well-defined cohort of 1006 patients with relapsing-remitting and secondary progressive MS from Australia. MS clinical outcome measures used in this study included age of onset, time interval from the first demyelinating event (FDE) to first relapse, and MS Severity Score (MSSS). Cerebral atrophy was assessed using a validated linear cerebral MRI measure, the intercaudate ratio (ICR), for 755 patients. In addition, cognitive testing using the symbol digit modalities test (SDT) was available for 869 patients.

## Materials and Methods

### Patients

The ethics committees involved were the Melbourne Health Human Research and Ethics Committee (HREC) (lead committee), Eastern Health HREC, Box Hill, Austin and Repatriation Hospitals HREC, Heidelberg, Barwon Health HREC, Geelong, St Vincent's Hospital HREC, Fitzroy, Ballarat Base Hospital HREC, Ballarat, Gippsland Base Hospital HREC, Sale (all in Victoria), Albury Base Hospital HREC, Albury, (NSW) and the Tasmania Health and Medical HREC (Tasmania). The ethics committees approved the project and signed informed consent was obtained from all participants.

A total of 1006 patients with relapsing-remitting (RRMS) and secondary progressive MS (SPMS) were recruited from Victorian and Tasmania in South-Eastern Australia between 2002 and 2004 [4], [15], [16].

All of the patients were assessed by six MS specialist neurologists. Clinical information including the age of onset, sex, time interval between first demyelinating event and first relapse, number of relapses and disease course of patients was collected. Disability in patients was determined using the Kurtzke Expanded Disability Status Scale (EDSS) The EDSS is a 10-point disease severity score with half-point increments which itself is derived from 9 ratings for individual neurological domains, including visual, brainstem, motor, sensory, cerebellar, bladder/bowel and cognitive function, and walking distance [17]. The EDSS was used in combination with disease duration to calculate the MS Severity Score (MSSS) with reference to the global MSSS table. The MSSS table essentially ranks individuals from lowest EDSS to highest EDSS for a given disease duration, and expresses this as a decile rank between 0 (least affected) and 10 (most severely affected) [18]. Cognitive disability was also assessed in 869 patients using the symbol digit modalities test (SDT) of the Wechsler Adult Intelligence Scale-revised [19]. SDT scores were adjusted for disease duration.

Age of onset was defined as the age at which the patient experienced their first demyelinating event and the interval between first demyelinating event and first relapse was determined from patient recall facilitated by a detailed questionnaire (provided to participants prior to the clinical assessment) and confirmed by medical notes (obtained wherever possible from their treating physician). Based on the country of birth of the participant's grand parents, the subjects were predominantly of Northern European ancestry, the majority were of Anglo Celtic (73%) or Southern European descent (16%). The remainder were of Western European (5%), Eastern European (4%), Scandinavian (1%) or other (1%) descent.

### Genotyping

DNA was collected and extracted from blood samples using either the Nucleon Genomic DNA Extraction Kit (GE Lifescience) or phenol-chloroform extraction. The majority of SNPs were genotyped at the Broad Institute for Genotyping and Analysis (http://www.broad.mit.edu/gen_analysis/genotyping/) with Sequenom's MassARRAY platform (San Diego, CA, USA) using the iPlex SNP assay design system [4]. However the SNPs rs6074022 and rs703842 were genotyped or imputed in a subset of 898 RRMS and SPMS patients as part of a genome-wide association study [5], either in the GWAS phase (Illumina 370CNV arrays, n = 581) or in the replication phase (Sequenom MassARRAY platform, n = 317). Presence or absence of the HLA-DR15 allele was determined from HLA-DRB1 typing performed previously in 954 of the cases using sequence-specific oligonucleotide hybridization and nucleotide sequencing [6].

### MRI assessment

Existing MRI scans were retrieved for measurements of linear markers of brain atrophy. Scans were available for 755 of the patients.

The clinical scans were performed at multiple centres and a variety of protocols were used, but generally the T1-weighted axial scans were of 5mm thickness with either no gaps (inter-leaved) or 2.5mm gaps. Intercaudate distance (ICD) was described as the minimum distance between the medial borders of the head of the caudate nuclei as previously described and validated [20]. The intercaudate ratio (ICR) was calculated as a fraction of the ICD to the transverse skull diameter (TSD) where the TSD was defined as the minimum distance separating the inner borders of the skull at the level of the most rostral part of the frontal horns. ICD and TSD were obtained from the same MRI slice, specifically the most caudal axial T1-weighted slice on which the frontal horns were at maximal width. ICR was log-transformed to give a more normally-distributed phenotype, and adjusted for the disease duration.

### Statistical Analysis

Five measurements of disease severity (MSSS, age of onset, time between the FDE and first relapse, duration-adjusted SDT and duration-adjusted log(ICR)) were tested for association with SNPs in *CLEC16A* (rs6498169), *IL2RA* (rs2104286), *IL7R* (rs6897932), *CD58* (rs12044852), *RPL5* (rs6604026), *CD40* (rs6074022) genes and at Chr12q13–14 (rs703842). Statistical analysis was performed using Stata 10 statistical software (StataCorp). Testing was performed by regression of continuous severity variables on the number of minor alleles carried by patients. All traits were approximately normally distributed except for MSSS (uniformly distributed by definition). For nominal genotype-phenotype associations (p<0.05), further analyses were performed, stratifying by sex and presence or absence of HLA-DR15. In addition, mean values and 95% confidence intervals were calculated for each severity variable, stratified by genotype. For variables associated with disease duration (ICR and SDT), means and confidence intervals were standardized to the mean disease duration (13.4 years) using the Stata ‘lincom’ command. The statistical methodology used in this paper has been previously published [16].

## Results

There were 1006 patients in total, of whom 78% were female. Patient demographic information is displayed in Table 1. Approximately 57% of patients carried at least one copy of HLA-DR15. Mean values of each of the MS severity variables were similar in each of the categories of females and males, and DR15-positive and DR15-negative individuals with the exception of age of onset, which was lower in DR15-positive patients, as expected in a cohort of northern European origin [21]. The ranges for each variable were; MSSS 0.05–9.82, Age of onset 7-65yrs, time between first demyelinating event (FDE) and first relapse 0–52 yrs, SDT 0–80, log(ICR) −1.099 to −2.996, means and standard deviations for these variables are shown in Table 1. Table 2 shows means and 95% confidence intervals for each of the severity measures stratified by genotype at each of the tested MS susceptibility SNPs. Due to the continuous nature of sample collection, some individuals were not typed for rs6074022 or rs703842, for which 897 and 898 genotypes were available, respectively, and for other assays, genotyping results were rejected in up to 5% of reactions when SNPs were called ambiguously.

**Table 1:** Demographic and clinical data for 1006 MS patients with RRMS or SPMS.

Metric	Total	Females	Males	HLA-DR15-	HLA-DR15+
Patients	1006	781	225	414	540
Age of onset	31.20 (9.84)	31.15 (9.74)	31.36 (10.19)	32.08 (10.18)	30.46 (9.56)
Time between FDE and 1^st^ relapse (N = 1002)*	5.13 (6.53)	5.01 (6.22)	5.56 (7.51)	5.25 (6.31)	5.03 (6.29)
MSSS	4.12 (2.62)	3.96 (2.54)	4.70 (2.79)	4.10 (2.58)	4.20 (2.63)
Symbol Digit Test (N = 850)	41.44 (12.69)	43.09 (12.26)	35.51 (12.49)	41.43 (12.32)	41.21 (13.06)
Log (ICD/TCD) ratio (N = 755)	−2.08 (0.30)	−2.11 (0.30)	−1.99 (0.26)	−2.08 (0.28)	−2.08 (0.31)

All results shown as: Mean (standard deviation).

*FDE = first demyelinating event.

HLA-DR15 status was available for 954 patients.

**Table 2:** MS disease severity metrics stratified by genotype.

					MSSS			Age of onset			Time 1&2			SDT (at mean duration)			Log((ICR) (at mean duration)		
Gene	SNP	Minor Allele	Alleles	N	Mean	CI		Mean	CI		Mean	CI		Mean	CI		Mean	CI	
CLEC16A	rs6498169	G	0	355	4.147	3.880	4.413	31.242	30.231	32.253	4.957	4.300	5.615	41.903	40.564	43.242	−2.092	−2.126	−2.058
			1	489	4.120	3.882	4.358	31.012	30.120	31.905	5.281	4.682	5.880	40.753	39.640	41.867	−2.060	−2.089	−2.030
			2	112	4.156	3.677	4.634	32.152	30.335	33.968	4.866	3.678	6.054	42.473	40.243	44.702	−2.096	−2.156	−2.036
IL2RA	rs2104286	G	0	563	4.160	3.938	4.382	31.020	30.187	31.852	5.062	4.530	5.595	41.315	40.266	42.363	−2.072	−2.099	−2.045
			1	378	4.008	3.749	4.268	31.603	30.623	32.584	5.221	4.578	5.863	41.749	40.481	43.016	−2.090	−2.123	−2.056
			2	38	4.810	3.975	5.644	29.474	26.408	32.540	7.053	3.693	10.412	40.137	36.340	43.934	−2.041	−2.145	−1.937
IL7R	rs6897932	T	0	542	4.105	3.887	4.324	31.059	30.200	31.918	5.293	4.746	5.839	41.259	40.213	42.305	−2.077	−2.105	−2.049
			1	356	4.190	3.914	4.466	31.225	30.247	32.202	4.992	4.279	5.704	41.270	39.939	42.600	−2.072	−2.106	−2.038
			2	57	4.024	3.313	4.736	32.825	30.181	35.468	4.246	2.939	5.553	43.752	40.238	47.267	−2.089	−2.177	−2.000
CD58	rs12044852	A	0	6	3.932	0.873	6.990	33.167	21.856	44.478	5.833	−0.301	11.968	44.557	34.363	54.752	−1.943	−2.223	−1.663
			1	194	4.040	3.671	4.409	30.727	29.242	32.212	5.440	4.528	6.353	41.421	39.658	43.184	−2.100	−2.146	−2.053
			2	758	4.168	3.981	4.355	31.302	30.610	31.995	5.050	4.581	5.520	41.396	40.494	42.297	−2.069	−2.093	−2.045
EV15/RPL5	rs6604026	C	0	464	4.125	3.885	4.364	31.086	30.163	32.010	5.577	4.939	6.215	41.926	40.774	43.077	−2.073	−2.103	−2.043
			1	419	4.177	3.925	4.429	31.308	30.386	32.230	4.766	4.176	5.355	40.782	39.571	41.993	−2.068	−2.100	−2.036
			2	74	3.938	3.335	4.542	31.527	29.267	33.787	4.162	2.921	5.403	41.903	39.007	44.799	−2.134	−2.211	−2.057
CD40	rs6074022	C	0	456	4.005	3.770	4.240	31.167	30.283	32.050	5.382	4.777	5.988	41.687	40.573	42.801	−2.072	−2.100	−2.044
			1	357	4.154	3.881	4.427	30.608	29.579	31.636	5.425	4.694	6.157	40.543	39.294	41.793	−2.071	−2.104	−2.039
			2	84	4.518	3.966	5.071	31.381	29.212	33.549	4.393	3.144	5.642	37.833	35.242	40.423	−2.068	−2.133	−2.003
Chr12q 13−14	rs703842	C	0	483	4.002	3.773	4.231	30.911	30.032	31.790	5.174	4.582	5.767	41.175	40.092	42.258	−2.065	−2.093	−2.037
			1	351	4.318	4.047	4.588	31.185	30.177	32.194	5.493	4.801	6.184	40.112	38.852	41.371	−2.079	−2.110	−2.047
			2	64	3.860	3.177	4.543	30.516	27.953	33.078	5.219	3.305	7.133	42.798	39.775	45.821	−2.071	−2.142	−1.999

Symbol digit test (SDT) and intercaudate ratio (ICR) scores have been standardised to the mean duration of 13.4 years. MSSS is the multiple sclerosis severity score. ICR is the intercaudate distance divided by the transverse skull diameter. Time 1& 2 is the time between the first demyelinating event and first relapse.

The effects of risk SNPS in this cohort of patients was analysed against five MS severity variables, and the results of this analysis are expressed as estimated change in disease severity variable for each additional minor allele (Table 3).

**Table 3:** Tests of association between SNPs and MS disease severity metrics.

						MSSS		Age of onset		Time 1&2		SDT		Log(ICR)	
Gene	SNP	Minor Allele	Alleles	MAF	N	Coef	p-val	Coef	p-val	Coef	p-val	Coef	p-val	Coef	p-val
CLEC16A	rs6498169	G	A/G	0.371	983	−0.020	0.877	0.207	0.669	−0.029	0.928	−0.228	0.710	0.010	0.536
IL2RA	rs2104286	G	A/G	0.232	979	0.022	0.879	0.088	0.874	0.465	0.207	0.031	0.964	−0.005	0.775
IL7R	rs6897932	T	C/T	0.246	955	0.024	0.866	0.515	0.327	−0.410	0.239	0.569	0.409	−0.001	0.951
CD58	rs12044852	A	C/A	0.108	958	−0.210	0.845	1.982	0.624	0.704	0.793	3.156	0.545	0.111	0.435
EV15/RPL5	rs6604026	C	T/C	0.296	957	−0.028	0.838	0.221	0.663	−0.754	**0.024**	−0.524	0.422	−0.011	0.507
CD40	rs6074022	C	T/C	0.293	897	0.215	0.102	−0.148	0.765	−0.289	0.396	−1.529	**0.016**	−0.006	0.716
Chr12q13–14	rs703842	C	T/C	0.267	898	0.109	0.428	0.022	0.966	0.160	0.654	−0.013	0.985	−0.013	0.444

MAF is the minor allele frequency. Fitted coefficients (coef) give the estimated change in disease severity metric for carriage of each additional minor allele. P-values arise from tests of whether these fitted coefficients differ significantly from zero. Symbol digit test (SDT) and intercaudate ratio (ICR) scores have been adjusted for disease duration. MSSS is the multiple sclerosis severity score. ICR is the intercaudate distance divided by the transverse skull diameter. Time 1& 2 is the time between the first demyelinating event and first relapse.

No associations were found between MS-risk-associated SNPs and MSSS, age of onset, or ICR. We observed a trend towards association between the *RPL5* SNP rs6604026 and the time between FDE and first relapse (p = 0.024), and between the *CD40* SNP rs6074022 and the duration of illness adjusted SDT score (p = 0.016). For both of these associations the minor, disease-associated allele was associated with more severe disease, however these trends were not significant after applying the Bonferroni correction for multiple testing, the threshold for which was p = 0.05/35 = 0.0014.

To account for the possibility that particular subgroups of patients might exhibit different genotype-phenotype effects, we stratified the effect of *RPL5* (rs6604026) genotype on the time between FDE and first relapse, and the effect of *CD40* (rs6074022) genotype on the SDT score by either sex or HLA-DR15 status.

In the subgroup analysis, the effect of *RPL5* (rs6604026) on the time between FDE and first relapse was very similar between groups. There was no significant interaction or trend between rs6604025 and sex (p = 0.915) or between rs6604025 and HLA-DR15 status (p = 0.650) (Table 4). When *CD40* (rs6074022) results were stratified by sex, there was a weak trend towards a stronger effect in males than females (coefficients −3.1247 and −1.1795 respectively), and a weak trend towards a stronger effect in HLA-DR15 negative individuals than HLA-DR15 positive individuals (coefficients −1.8818, and −0.9834 respectively), but these trends were not significant (interaction p-values 0.475 and 0.224, respectively) (Table 4).

**Table 4:** Nominally significant results (p<0.05) stratified by sex and DR15 genotype.

	EV15/RPL5		CD40	
	rs6604026		rs6074022	
Metric	Time 1&2		SDT	
Stratification	Coef	p-val	Coef	p-val
Overall	−0.754	0.024	−1.529	0.016
female	−0.765	0.033	−1.1795	0.086
male	−0.679	0.412	−3.1247	0.025
DR15−	−0.844	0.100	−1.8818	0.054
DR15+	−0.540	0.219	−0.9834	0.263
Interaction with sex	0.086	0.915	0.93613	0.475
Interaction with DR15	0.304	0.650	−1.7919	0.224

P-values arise from tests of whether these fitted coefficients differ significantly from zero. Time 1& 2 is the time between the first demyelinating event and first relapse. Symbol digit test (SDT) scores have been adjusted for disease duration.

## Discussion

In this study, we investigated whether seven recently identified MS risk-associated SNPs were acting as modifiers of severity on any of five markers of MS severity in a large cohort of relapsing-remitting and secondary progressive MS patients from south-eastern Australia. We chose to investigate these associations using an additive, allele-load model of inheritance, rather than recessive and dominant models, to reduce multiple testing. We found no evidence that any of the tested SNPs modified the clinical phenotypes of MSSS, age of onset or the time between FDE and first relapse. We also did not detect an effect of the risk SNPs on cerebral atrophy as measured by the ICR, and no evidence of effects on cognitive function as measured by the SDT. We have previously reported that none of the seven risk-associated SNPs tested here are determinants of relapsing versus primary progressive phenotypes [5]. Similarly, a recent genome-wide association study of 1000 people with MS [22] investigated SNP markers associated with age of onset, MSSS, cerebral atrophy and T2 lesion load, and none of the seven risk SNPs assessed in this study were reported as showing strong trends of association with the tested phenotypic variations.

In our study, MS risk-associated SNPs in *CD40* and *RPL5* showed weak trends towards association with specific disease severity measures, namely with ICR and time between FDE and first relapse, respectively. These associations were not significant after appropriate correction for multiple testing. As these two SNPs may potentially only be associated with disease severity in subgroups of patients, the subjects were stratified on two known risk factors, sex and HLA-DR15 status. There was weak, non-significant evidence that the *CD40* SNP has a stronger effect on ICD in males and HLA-DR15 negative individuals. The observed lack of effect of *CD40* on phenotype is consistent with a previous study of disease severity (EDSS), which used an extremes of severity approach and could not detect an association between severity and *CD40* genotype [23].

This study only examined associations between the identified risk SNP genotypes and MS disease severity. The currently identified risk SNPs may be causative, or they may be in linkage disequilibrium with as yet unidentified SNPs in the same or neighbouring candidate genes, which could potentially carry stronger associations with MS. Fine mapping or sequencing of genetic regions near the currently identified risk SNPs will probably refine the current MS genetic associations and could also change the results of our analysis of genetic modulation of MS disease severity.

In the analyses utilising the full sample (eg MSSS outcomes), our study had 80% power to detect associations with SNPs that account for at least 1.6% of a trait's variance, Due to missing data, power was reduced in some analyses. For the smallest sub-sample (MRI outcomes) we had 80% power to detect associations with SNPs that account for at least 2.1% of variance after correction for the 35 tests in Table 3 (significance level of p = 0.0014). We had 80% power to detect nominally significant associations (p = 0.05) with SNPs that account for at least 0.8% of a trait's variance (full sample) or at least 1.0% of variance (individuals with MRIs).

Given that our study did not show significant associations between individual risk SNPs and MS severity, we would not expect interactions between multiple SNPs to greatly influence MS severity. However, although the cohort size of this study is large, it still does not permit formal testing of interaction hypotheses, as even interaction tests between any two of the seven risk SNPs would result in 42 interactions to be tested, and the power of such a study would be very low.

Interaction tests between treatment exposures and risk SNPs for association with MS severity are also difficult to conduct because of power issues. However, at the time of clinical assessment, 71% of the study cohort was on treatment with one of four disease-modifying drugs available at the time. If there were a large, SNP-specific treatment effect, we would expect to detect it in this study as the particular SNP would, in this case, be a characteristic of the individuals with reduced MS severity.

Few allelic variations associated with MS severity have been found, and the only one to be confirmed in multiple studies is the association between increasing allele load of HLADR15 and lower age at MS onset [2], [7], [8], [9], [10]. The effect of HLA-DR15 on other markers of disease severity is contentious. While some studies have shown no effect [24], [25], other studies have shown that HLA-DR15 influences several other markers of MS disease severity including the number of lesions at presentation [13], normalized brain volume, cognitive function [26] and even the type of early clinical manifestation of MS [27]. Of the three alleles of the apoE gene, ApoE4 has been reported to be associated with worse outcome, but large studies, including our own [16]and a recent meta-analysis [28] did not confirm any of these effects. Other, as yet unreplicated disease-modifier SNPs have been reported. For example, a SNP in IL-1β, which leads to higher expression of the protein, has been reported to be associated with more benign disease, as assessed by duration-adjusted EDSS [29]. Two different polymorphisms in the promoter region of the matrix metalloproteinase 9 (MMP9) gene are associated with higher MMP9 expression and, in MS cases, were reported to lower the age-of-onset [30], [31] however this effect was not replicated in another study [32]. Polymorphisms in the interleukin 4 gene and its receptor (IL4R) have also been shown to be associated not only with MS susceptibility [33], [34], [35] but also with a primary progressive course [36].

More recently, a genome-wide phenotype-genotype study using gene-ontology techniques in patients with relapsing-remitting MS [22] reported that the gene function categories “antigen processing and presentation” and “CNS development” were enriched in MS susceptibility whereas the categories of “axon guidance” and “glutamate signalling processes” were implicated in phenotypes of CNS damage, namely, T2 lesion load and brain volume.

The metrics in this study were chosen because they could be assessed in a large cohort of people with MS, and thus, it is possible that different measures could detect changes that could not be assessed here. SDT, for example, is a good screening tool for cognitive functional ability in MS, but if the various SNPs are associated with a regional deficit, or a specific functional change, then such an effect would not be detected in our study. Likewise, ICR is a validated measure of overall brain atrophy, but if potential modulating effects of the MS associated SNPs were brain-region specific, then the change might not be detected in this study. Additionally, it has been shown that aspects of MS disease phenotype are population-specific or stratify on the basis of ethnic origin. For example, in British and Scandinavian cohorts the HLA-DR2 allele imparts an earlier age of onset of disease [2], [7], [8], [9], [10], whereas in a cohort from the USA no effect was observed [11]. Beyond this, the potential interactions between disease associated SNPs and environmental factors may be different in cold climates than from tropical ones. As such, our results are particularly applicable to an MS population of Caucasian of predominantly British origin, resident in the temperate mid-latitudes (36°–43°S).

In this population, our study provided no evidence that MS severity or progression was altered by recently confirmed risk alleles in MS sufferers.
